# Development of maizeSNP3072, a high-throughput compatible SNP array, for DNA fingerprinting identification of Chinese maize varieties

**DOI:** 10.1007/s11032-015-0335-0

**Published:** 2015-05-31

**Authors:** Hong-Li Tian, Feng-Ge Wang, Jiu-Ran Zhao, Hong-Mei Yi, Lu Wang, Rui Wang, Yang Yang, Wei Song

**Affiliations:** Maize Research Center, Beijing Key Laboratory of Maize DNA Fingerprinting and Molecular Breeding, Beijing Academy of Agriculture and Forestry Sciences, Shuguang Garden Middle Road No. 9, Beijing, 100097 China

**Keywords:** Maize variety, Variety identification, Single nucleotide polymorphism (SNP), DNA fingerprinting, Molecular breeding

## Abstract

Single nucleotide polymorphisms (SNPs) are abundant and evenly distributed throughout the maize (*Zea mays* L.) genome. SNPs have several advantages over simple sequence repeats, such as ease of data comparison and integration, high-throughput processing of loci, and identification of associated phenotypes. SNPs are thus ideal for DNA fingerprinting, genetic diversity analysis, and marker-assisted breeding. Here, we developed a high-throughput and compatible SNP array, maizeSNP3072, containing 3072 SNPs developed from the maizeSNP50 array. To improve genotyping efficiency, a high-quality cluster file, maizeSNP3072_GT.egt, was constructed. All 3072 SNP loci were localized within different genes, where they were distributed in exons (43 %), promoters (21 %), 3′ untranslated regions (UTRs; 22 %), 5′ UTRs (9 %), and introns (5 %). The average genotyping failure rate using these SNPs was only 6 %, or 3 % using the cluster file to call genotypes. The genotype consistency of repeat sample analysis on Illumina GoldenGate versus Infinium platforms exceeded 96.4 %. The minor allele frequency (MAF) of the SNPs averaged 0.37 based on data from 309 inbred lines. The 3072 SNPs were highly effective for distinguishing among 276 examined hybrids. Comparative analysis using Chinese varieties revealed that the 3072SNP array showed a better marker success rate and higher average MAF values, evaluation scores, and variety-distinguishing efficiency than the maizeSNP50K array. The maizeSNP3072 array thus can be successfully used in DNA fingerprinting identification of Chinese maize varieties and shows potential as a useful tool for germplasm resource evaluation and molecular marker-assisted breeding.

**Electronic supplementary material:**

The online version of this article (doi:10.1007/s11032-015-0335-0) contains supplementary material, which is available to authorized users.

## Introduction

Maize (*Zea mays* L.) is an important crop widely grown throughout the world for food, feed, and fuel production. In China, maize is the top-ranked crop in terms of cultivated area and total yield and plays a key role in the country’s agricultural economic structure. As a hybrid crop, maize is additionally a model plant species for genetic studies because of its high recombination rate and rich genetic diversity. Since the 1980s, the number of maize varieties in China has steadily increased. As of 2013, the total number of varieties approved by national and provincial governments is 6291, including 503 approved by the national government. Because thousands of maize samples are inspected annually in China (Yang et al. 2014), the large number of maize varieties complicates variety management. At the same time, convergence of breeding resources and patterns has resulted in a gradual narrowing of the maize germplasm genetic base, causing a negative effect on breeding and seed production. The identification of new and existing varieties has consequently become challenging. Traditional field identification and protein electrophoresis cannot meet the need for rapid and accurate identification because of their limited ability to distinguish varieties and the long turnaround time required. DNA fingerprinting technology has become an important approach for distinguishing maize varieties, as this technique is rapid, accurate, and independent of the environment.

Over the past two decades, several different DNA marker technologies, including those based on restriction fragment length polymorphisms, inter-simple sequence repeats (ISSRs), amplified fragment length polymorphisms, simple sequence repeats (SSRs), and single nucleotide polymorphisms (SNPs), have been widely applied in research areas such as DNA fingerprinting of varieties, genetic diversity analysis, association studies, and molecular marker-assisted breeding (Nandakumar et al. 2004; Coombs et al. 2004; Wang et al. 2011a; Barcaccia et al. 2003; Garcia et al. 2004; Clerc et al. 2005; Lu et al. 2009; Semagn et al. 2012; Khampila et al. 2008; Weng et al. 2011; Lu et al. 2011; Chen et al. 2011; Chai et al. 2012; Thomson et al. 2012). DNA fingerprinting refers to the identification of the different compositions, orders, and lengths of DNA sequences among varieties, which, like human fingerprints, are specific. Compared with other markers, SSR and SNP markers have the advantages of codominant inheritance and known chromosomal location and can be used in high-throughput analyses. Consequently, the International Union for the Protection of New Varieties of Plants (UPOV 2010, 2011) recommends SSRs and SNPs as preferred markers for DNA fingerprinting and database construction.

SSR markers have been used for variety identification for more than 10 years because of their high discriminatory power and associated relatively well-developed experimental techniques, which are easily performed without expensive instrumentation. With the development of new technologies and increased identification requirements, however, SSR markers have been shown to possess some disadvantages. For example, the throughput of locus processing cannot be easily increased, and data comparison and integration between different detection platforms are difficult. Compared with SSRs, SNPs offer several advantages. They display a higher and more even distribution density in the genome; in maize, for example, SNP loci occur every 44–75 bp (Gore et al. 2009), whereas SSR loci are present approximately every 8 kbp (Wang et al. 1994). In addition, SNPs are bi-allelic, making them easy to read, compare, and integrate between different data sources, and they facilitate high-throughput processing. Finally, SNP loci are more likely to be distributed within a gene region associated with a phenotype. With the recent publication of whole-genome sequences of crops such as maize and rice (Huang et al. 2009; Schnable et al. 2009; Lai et al. 2010; Jiao et al. 2012), numerous SNP loci have been developed (Gore et al. 2009; Chia et al. 2012; Chen et al. 2011) and various SNP genotyping platforms have been introduced. SNPs have become the most promising markers for DNA fingerprinting and database construction. A variety of SNP genotyping platforms are available, such as the high-throughput GoldenGate (Fan et al. 2003) and Infinium platforms (Steemers and Gunderson 2007), TaqMan by Life Technologies (Livak et al. 1995), and the KASPar platform (KBiosciences’ Competitive Allele-Specific PCR system). When a relatively small number (i.e., dozens) of SNP loci are to be assayed, a relatively flexible platform such as TaqMan, Sequenom, or KASPar is recommended; when more than 100 loci are available, the high-throughput GoldenGate or higher-throughput Infinium platform should be chosen. For DNA fingerprinting and database construction, platforms based on chip technology, such as GoldenGate and Infinium, are appropriate choices.

Numerous studies have focused on the development, evaluation, and application of SNP loci in maize. Many SNP markers have been developed by whole genome or transcriptome sequencing (Jones et al. 2009; Gore et al. 2009; Lai et al. 2010; Mammadov et al. 2010; Jiao et al. 2012; Chia et al. 2012). In addition, Infinium and GoldenGate platforms have been used to develop a variety of SNP arrays, such as the high-density SNP array maizeSNP50 (Ganal et al. 2011) that has been successfully applied to genome-wide association and quantitative trait locus (QTL) mapping studies (Weng et al. 2011; Wang et al. 2012). Moreover, various sized SNP arrays have been used to assess genetic diversity in maize (Yan et al. 2009; Lu et al. 2009; Yan et al. 2010; Hao et al. 2011; Semagn et al. 2012).

Although many SNP markers are available in maize, only a small percentage of polymorphic loci can be combined in an SNP array. Consequently, selection and evaluation of SNP sets are important steps in maize DNA fingerprinting. Fingerprint databases that have been constructed using only fixed locus sets are also valuable. The criteria used to select loci for variety identification are quite different from those used in research areas such as genetic diversity and association analyses. Previously reported SNP sets cannot be directly applied to maize variety identification. In this study, we consequently selected and evaluated SNP loci for maize DNA fingerprinting analysis from the maizeSNP50 array, which contains 56,110 SNPs, using Chinese maize varieties with broad genetic backgrounds. We also examined the stability of this SNP array through sample amplification and examined its discriminatory power, compatibility, and applicability to maize DNA fingerprinting identification.

## Materials and methods

### Plant materials and DNA extraction

A total of 96 samples were selected to evaluate the SNP markers, including 40 hybrids and 56 inbred individuals (Electronic Supplementary Material Table S1). The 40 hybrid samples included varieties with large planting areas, control varieties from different maize regional trial groups, and some specialized hybrids. The 56 inbred samples included the corresponding parents of the above hybrids and other elite inbred lines (22 triplets with their parents and the F_1_ generation), four groups of similar lines, and a group of doubled haploid lines. Total genomic DNA was extracted from 50 pooled leaf samples using the CTAB procedure according to Wang et al. (2011b). DNA quality and concentration were measured with a NanoDrop 2000 UV spectrophotometer (Thermo Scientific, MA, USA), and working solutions were prepared at a concentration of 100 ng/μL.

### maizeSNP50 SNP genotyping

The 96 DNA samples were analyzed using the maizeSNP50 BeadChip containing 56,110 SNP loci (Ganal et al. 2011). Raw data were obtained by scanning the chip with hybridized signals using an iScan instrument (Illumina). The genotype data from each sample were analyzed with GenomeStudio software (v2010; Illumina) using the maizeSNP50_B.egt cluster file.

### Selection of SNP loci

We first mapped 56,110 SNP loci to the maize B73 sequence and identified the physical location of each locus. SNP selection was performed using a three-step process. First, we selected candidate loci on the basis of GenomeStudio GenTrain scores. These scores can range from 0.00 to 1.00, reflecting the accuracy of the data: The higher the score, the more reliable the data. The GenTrain score of each SNP is calculated according to the following SNP genotype cluster characteristics: angle, dispersion, overlap, and intensity. Genotypes with lower scores are located further from the cluster center and have a lower reliability. From the 56,110 SNPs, a total of 35,894 (64 %) SNPs with GenTrain scores between 0.70 and 1.00 were designated as candidate loci. Second, 20,212 SNPs were selected on the basis of reproducibility, missing data rate, signal strength, and their utility for defining the three genotypes. If a sample data point fell outside of a shaded call region on the GenomeStudio SNP graph, it was treated as missing data. SNPs with a missing data rate of more than 5 % of sites were removed. As the three genotypes of an ideal SNP should have obvious boundaries on the graph and be easy to differentiate, any SNP having a shifted cluster or a non-obvious boundary was deleted. Third, the candidate loci were further screened based on copy number, minor allele frequency (MAF), and even distribution. SNPs with copy numbers greater than or equal to 2 or MAF values under 0.2 were deleted. The remaining loci were screened according to the physical map. The best SNP in each genic region was chosen on the basis of coding region priority and good experimental quality principles.

### The maizeSNP3072 array

The 4050 candidate SNPs were submitted to Illumina (http://www.illumina.com) to assess their designability score values based on the GoldenGate assay. The key factors influencing these scores were the accuracy and conservation of the DNA sequences located 100 bp upstream and downstream of the SNP site. A score higher than 0.6 indicated that the SNP had a relatively higher probability of success, and SNPs with scores below 0.4 were deleted. A total of 3072 SNPs were obtained. The probe pool was developed according to the flanking sequences of the 3072 SNPs, and the maizeSNP3072 array chip was ordered based on GoldenGate technology.

### Evaluation of the maizeSNP3072 array

The 96 samples were genotyped using the maizeSNP3072 array to verify the repeatability of the 3072 SNPs. To assess the stability and discriminatory power of the 3072 SNPs, 309 inbred lines, including 217 elite lines commonly used in China and 92 US samples, were genotyped using this array. The 217 Chinese inbred lines had a wide genetic background that included six heterotic groups: Tang-si-ping-tou (STPT), P, Improved Reid, Lancaster, Waxy, Landrace. In addition, 276 hybrid samples representing varieties approved by the Chinese Ministry of Agriculture were used to assess the effects of the 3072 SNPs on maize DNA fingerprinting and database construction.

### Data analysis

Cluster differentiation of the three possible genotypes (AA, BB, and AB) was performed for the 3072 SNPs based on genotype data from 22 triplets, 309 inbred lines, and 276 hybrids. Repeatability, miss rate, polymorphism, and variety-distinguishing efficiency of the 3072 SNPs were analyzed using the data from 96 selected samples, 309 inbred lines, and 276 hybrids. To assess the compatibility of the 3072 SNPs, we analyzed the genotype consistency of repetitive samples between Infinium and GoldenGate platforms. Polymorphism of each locus was analyzed based on the inbred-line data. The percentage of different loci was analyzed according to pairwise comparisons among 309 inbreds and 276 hybrids.

## Results

### Selection process for the 3072 SNPs

**Fig. 1:**
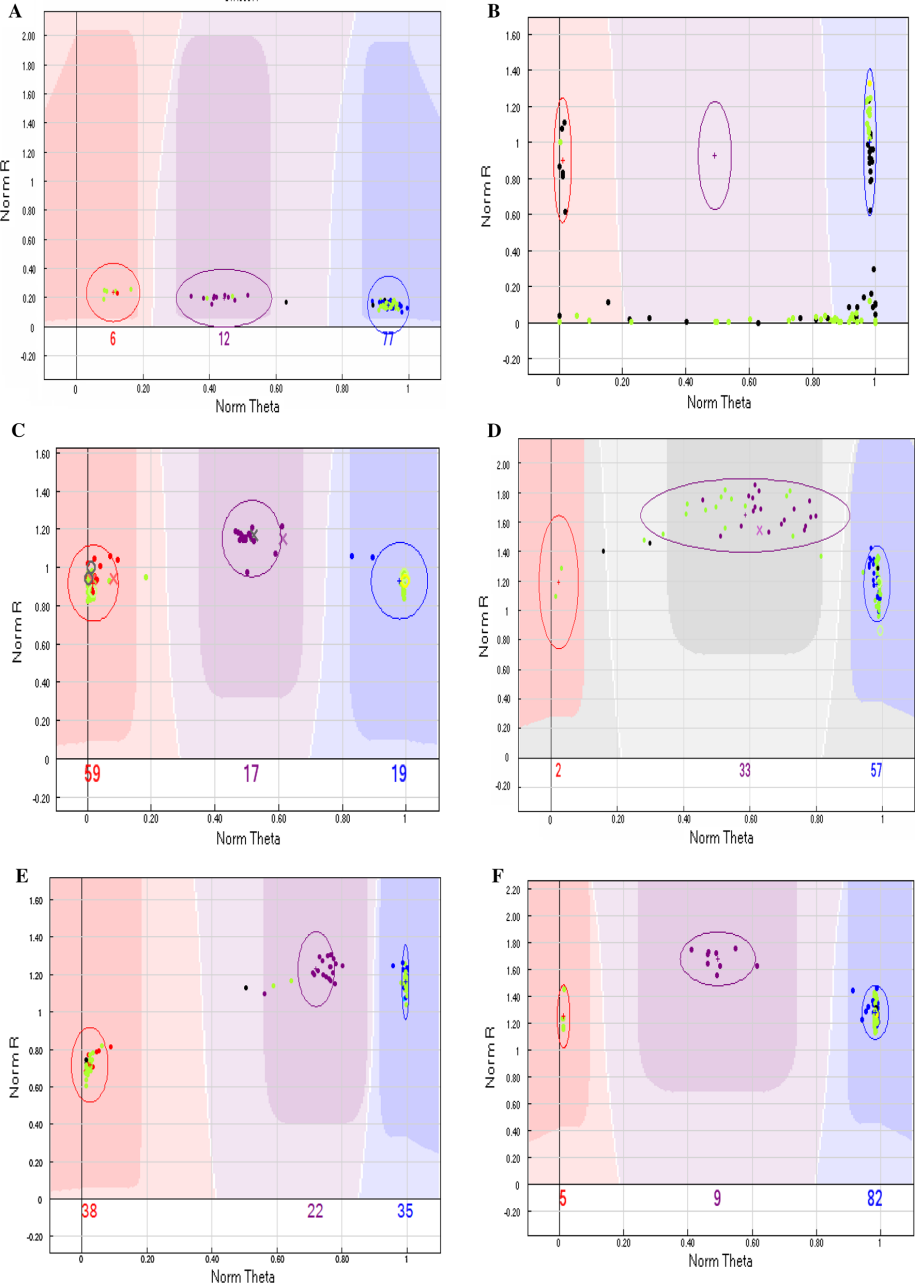
Different patterns of clustering of AA, AB, and BB genotypes based on GenomeStudio analysis. Each point is an actual call. The *three shaded areas* correspond to calculated limits, with *darker colors* indicating higher levels of confidence. *Ellipses* are used to adjust the position of the allele-calling areas. Three different genotypes are called: homozygous for allele A (*red*), heterozygous AB (*purple*), and homozygous for allele B (*blue*). Allele calls that fall in the lighter-colored areas in between or below these areas are set to “failed.” *Filled green circles* represent inbred-line data points. If any parent/F_1_ errors are found in the data, the F_1_ hybrid appears as an “X” and the parent as an “O.” **a** Only weak sample signals detected, **b** three clusters observed, but numerous failed samples (*at the bottom*) not called, **c** pedigree inconsistency exhibited by more than three parent/F_1_ combinations, **d** heterozygous genotypes shown by more than five inbred lines, **e** one or all three clusters shifted toward one side of the graph and **f** perfect genotyping locus in which the three genotypes fall into clearly defined clusters across all 96 samples. The *x*-axis represents normalized theta (angle deviation from pure A signal, with 0 indicating pure A signal and 1.0 representing pure B signal), and the *y*-axis corresponds to the distance of the point from the origin

GenTrain scores were calculated for 56,110 SNPs across the 96 selected samples. There were 16.2 % loci with scores less than 0.6, 19.8 % with scores between 0.6 and 0.7, and 64 % with scores higher than 0.7. The different patterns of AA, AB, and BB genotype calls obtained using GenomeStudio software are shown in Fig. 1. Five different types of loci were removed during the screening process: loci with weak signal values (Fig. 1a), loci with more than five failed data points (Fig. 1b), loci for which more than three parent/F_1_ triplets showed pedigree inconsistency (Fig. 1c), loci for which more than five inbred samples showed the AB genotype (Fig. 1d), and loci for which one or all three genotypes exhibited an obvious shift toward one side of the diagram (Fig. 1e). To be considered an ideal SNP, the three genotypes for that locus should fall into clearly defined clusters (Fig. 1f).

### Establishment of an accurate genotype-calling procedure through cluster differentiation

**Fig. 2:**
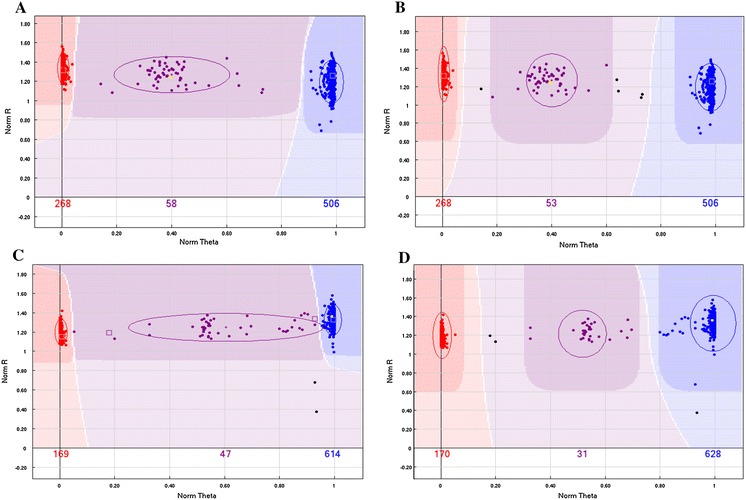
MaizeSNP3072 cluster file constructed to improve the genotyping efficiency of the 3072 loci. Samples with reproducibility errors appear as *squares*. **a**, **c** Automatic SNP calling using GenomeStudio software and **b**, **d** corrected SNP calling using a maizeSNP3072 cluster file

The raw data obtained from the iScan system were imported to GenomeStudio software for genotype analysis. SNPs were automatically called for AA, AB, and BB genotypes as shown in Fig. 2a, c. If a rare AB genotype was identified or some data points were shifted to one side, the automatic SNP calling frequently produced errors. To resolve this problem, we used parent/F_1_ triplets and inbred and hybrid samples to construct a high-quality standard cluster file, maizeSNP3072_GT.egt (Fig. 2b, d) which was used to define regions corresponding to the three genotypes. Characteristics of all sample data points for each SNP were visualized using SNP graphs in GenomeStudio. In these graphs, the x-axis represented normalized theta (angle deviation from pure A signal), with 0 corresponding to pure A signal, 1.0 to pure B signal, and 0.5 to the AB cluster theta mean (Figs. 1, 2). The cluster file was defined using these graphs according to the following criteria: (1) clear boundaries could be drawn between different genotypes; (2) the missing data rate was minimized; (3) center points of AA, AB, and BB clusters were positioned at 0, 0.5, and 1.0, respectively; and (4) center points could be shifted based on the actual evaluation results of the triplets and inbred and hybrid samples. The missing data rate for the 309 inbred lines, 6 %, was only 3 % when automatic analysis was performed using the combined cluster file.

### Characteristics of the maizeSNP3072 array

**Fig. 3:**
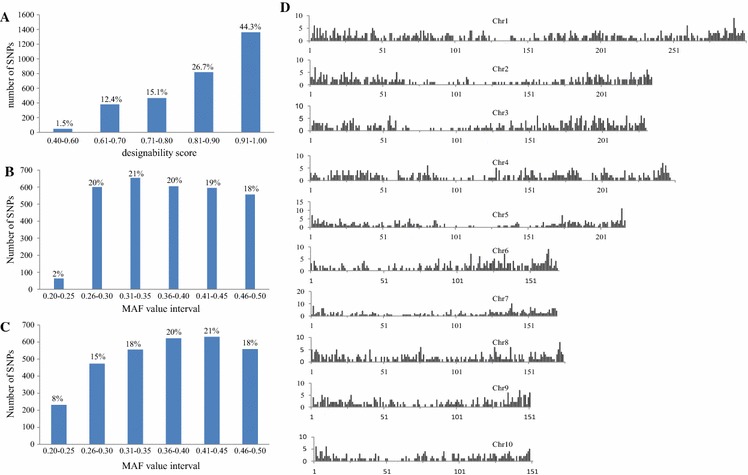
Design of the maizeSNP3072 array. **a** Numbers of single nucleotide polymorphisms (SNPs) with their corresponding designability scores for 3072 SNPs evaluated by Illumina, **b** MAF values of the 3072 SNPs based on data from 96 samples, **c** MAF values of the 3072 SNPs based on data from 309 inbred lines and **d** distribution of the 3072 SNPs on 10 chromosomes. The window size is 1000 kbp, the *x*-axis represents the order of the widows, and the *y*-axis corresponds to the number of SNP loci

Designability scores of candidate SNP loci, which were provided by Illumina, ranged from 0 to 1.0. A score higher than 0.6 indicates that an SNP has a relatively high probability of success when used in a GoldenGate assay, whereas a score below 0.4 indicates that the SNP is predicted to have a poor success rate. The designability score distribution of the 3072 SNPs was 1.5 % (0.40–0.60), 27.5 % (0.61–0.80), and 71 % (0.81–1.00) (Fig. 3a). Because of the wide genetic background of the 96 samples used to evaluate loci, the polymorphism rate of the 3072 SNPs exhibited little change when loci were assessed instead using 309 inbred lines. All MAF values were greater than 0.20, with an average of 0.37. The percentage of MAF values between 0.20 and 0.25 was relatively low (8 %), while percentages for other intervals were between 15 and 21 % (Fig. 3b, c).

Two 92-sample parallel experiments were performed to assess the compatibility of the 3072 SNPs on Infinium and GoldenGate platforms. The consistency percentage of the 3072 loci was 96.4 % based on 92-sample genotype data calling on the two platforms. The conversion rate of the 3072 SNPs between the two platforms was thus higher than 95 %. The parent-hybrid heritability of 22 combinations was analyzed using all SNPs that were scorable in each hybrid and its two parents (Electronic Supplementary Material Table S2). The parents had diverse genetic backgrounds that included Improved Reid, P, STPT, Luda Red Cob, SSS, NSSS, and landrace groups. Pedigree consistency values higher than 95 % were uncovered for 17 combinations, with a value of 100 % obtained for two combinations having B73 and/or Mo17 as parents. Consistency values below 95 % were observed in five combinations; this higher inconsistency was due to different seed sources for a hybrid and parents or the low purity rates of inbred lines such as Shen137 and Zong31.

Analysis of several similar inbred groups uncovered no differences among T877-series maize samples (Electronic Supplementary Material Table S1; ID nos. N91–N93). The genetic backgrounds of T877 lines were anticipated to be highly similar, as the series was produced by radiation-induced mutagenesis. Unlike T877 lines, Qi319, P25, and F349 lines were obtained through backcross breeding. Although some different loci were consequently uncovered in Qi319, P25, and F349 series (Table S1; ID nos. N84–N90), all genetic similarity values were greater than 97 %. To verify the ability of the 3072 SNPs to discriminate among maize varieties, we performed 37,950 and 47,586 pairwise comparisons among 276 hybrids and among 309 inbred lines. Differential locus percentages of between 5 and 70 % were observed in 99.9 % of these pairwise comparisons for both inbred and hybrid lines. The most frequent differential locus percentages uncovered were 50 % among inbred lines (in 4888 comparisons) and 60 % among hybrids (in 4216 comparisons) (Electronic Supplementary Material Figure S1).

### Distribution characteristics of the 3072 SNP loci

The physical distribution of the 3072 loci on the 10 maize chromosomes was determined using their mapped positions on the B73 genome sequence. Each chromosome was divided into 1000-kbp-sized windows, and the number of SNPs per window was counted (Fig. 3d). Almost all of the SNPs were found to be distributed evenly throughout the genome. SNPs were relatively sparse around centromeres and relatively abundant near telomeric regions (Fig. 3d). All 3072 SNPs were in genic regions, where they were distributed in exons (43 %), promoters (21 %), 3′ untranslated regions (UTRs; 22 %), 5′ UTRs (9 %), and intron regions (5 %).

### Comparative analysis of maizeSNP3072 and maizeSNP50K arrays

**Fig. 4:**
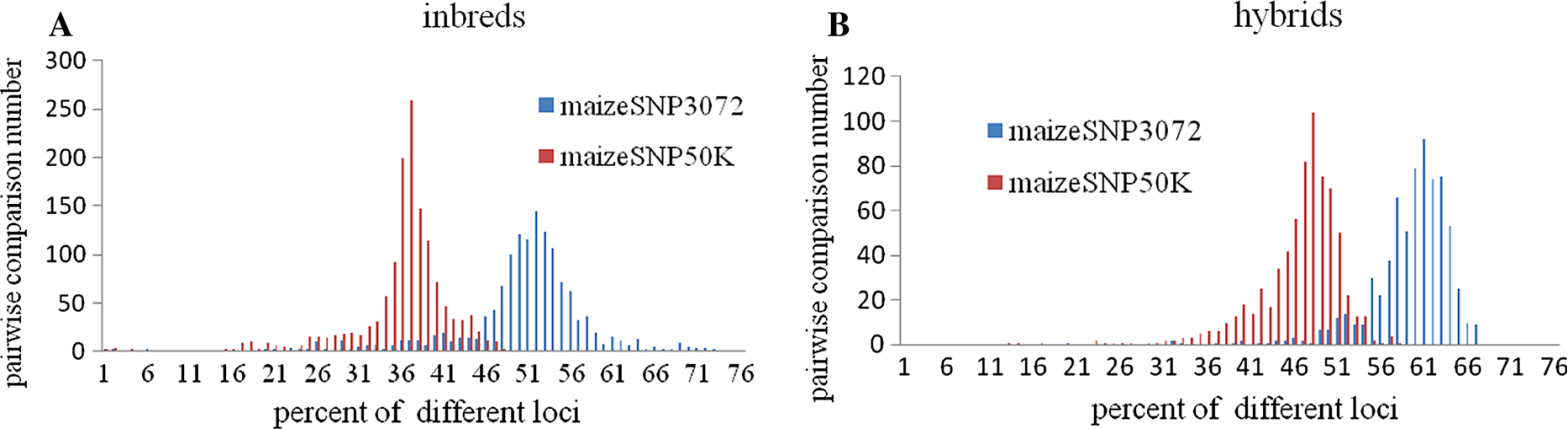
Distribution of different locus percentages obtained by pairwise comparative analysis of evaluated samples based on 3072 and 56,110 SNPs. **a** Comparison using genotyping data from 53 inbred lines. **b** Comparison using genotyping data from 38 hybrids

The performance of maizeSNP3072 and maizeSNP50K arrays was compared using the 96 samples evaluated in this study (Table 1; Fig. 4). On the Chinese materials, the 3072SNP array showed a better marker success rate and higher average MAF values, evaluation scores, and variety-distinguishing efficiency than the maizeSNP50K array. Differences in the distinguishing efficiency of the two arrays are shown in Fig. 4 for Chinese inbred and hybrid samples. Differential locus rates among inbreds ranged from 15 to 72 % (average of 49 %) and 12 to 52 % (average of 35 %) for 3072 and 56,110 SNPs, respectively; among hybrids, the corresponding rates were 16 to 66 % (average of 58 %) and 12 to 57 % (average of 45 %).

**Table 1:** Comparative analysis of maizeSNP3072 and maizeSNP50K chips based on data from 3072 and 56,110 single nucleotide polymorphisms in 96 evaluated maize samples

Comparative item	MaizeSNP3072	maizeSNP50K
Marker success rate	94 %	67 %
Average MAF value	0.37	0.17
GenTrain score (0.6)^a^	100 %	84 %
GenTrain score (0.7)^a^	89 %	64 %
Variety-distinguishing efficiency^b^	Inbred (49 %), hybrid (58 %)	Inbred (35 %), hybrid (45 %)

^a^Percentage of loci with GenTrain scores >0.6 or 0.7

^b^Average differential locus rate

## Discussion

### SNP array development and characterization for maize DNA fingerprinting

Fixed SNP sets are preferred for maize DNA fingerprinting and database construction. SNP marker development is well underway in maize, with numerous SNPs listed in databases such as Panzea and MaizeGDB. Not all SNPs are suitable for DNA fingerprinting, however, and some loci do not meet array chip design requirements. The selection of fixed SNP locus sets with high discriminatory ability, good stability, and even distribution is thus the most important step in SNP marker-based fingerprinting research. To construct an SNP array for maize DNA fingerprinting, a set of evaluation materials with a broad genetic basis, reasonable SNP selection principles, and an accurate cluster genotyping file are required. Polymorphism bias will be present if the genetic background of the selected materials is concentrated. In addition, maize DNA fingerprinting must be able to differentiate among hybrids quickly and accurately. Consequently, representative hybrids must be selected to validate the variety-discriminatory efficiency and heterozygous calling accuracy of candidate SNPs. Common assessment indices for selecting a set of SNPs include repeatability, discriminatory power, uniformity of distribution, and conservatism of flanking sequences. Automatic and accurate genotype calling also are quite important. To ensure that three genotype clusters can be easily distinguished, the selected SNP should be a single-copy locus, and both inbred and hybrid lines should be used to evaluate cluster independence and stability. Automatic SNP calling using GenomeStudio software is sometimes prone to mistakes, especially when a rare AB genotype cluster is present. To improve the accuracy and efficiency of genotype calling, a standard genotyping cluster file based on the characteristics of each SNP should therefore be established.

### Comparison of published SNP arrays and the maizeSNP3072 array

Published SNP genotyping arrays include the high-density maizeSNP50 array (Ganal et al. 2011), a 768-SNP array reported by the Pioneer Co. for commercial maize resource identification (Jones et al. 2009), a 1536-SNP array used for germplasm resource assessment (Lu et al. 2009; Yan et al. 2010; Semagn et al. 2012), and an SNP array used in an association study (Yan et al. 2009). SNP selection criteria for DNA fingerprinting are different from those used for association and QTL analyses (Yan et al. 2009), with the latter focused on loci related to objective characters. In this study, we selected 3072 SNPs using inbred and hybrid lines as evaluation materials on the basis of their stability, genotype-calling accuracy, discriminatory power, copy number, and evenness of distribution.

The number and sources of SNPs initially employed in this study were largely different from those used to produce the 768- and 1536-SNP sets described above. In particular, 3072 SNPs were selected from 56,110 SNP loci in our study, whereas approximately 2000 initial loci were used to construct the 768- and 1536-SNP sets. The 768- and 1536-SNP sets were selected primarily from PHM loci (developed by Pioneer) and PZA loci in the Panzea database, of which only a small proportion were candidate loci in the current study. The 3072 SNP loci thus have few similarities with the 768 and 1536 loci. In conclusion, compared with the previously reported SNP arrays, the maizeSNP3072 set is more suitable for Chinese maize variety DNA fingerprinting and database construction.

### Selection of SNPs compatible with Infinium and GoldenGate platforms

Although many types of SNP genotyping platforms exist, such as GoldenGate, Infinium, TaqMan, and KASPar, not all SNPs are transferable across different platforms. A success rate of approximately 89 % has been obtained on GoldenGate and Infinium platforms (Mammadov et al. 2012). Although both platforms are based on bead-chip technology, they differ somewhat in regard to probe design, reagents, and experimental processes. Some SNPs may therefore not be transferable across the two platforms. The SNPs identified in this study, which were evaluated using Infinium and GoldenGate platforms, had a conversion rate between the two platforms of over 95 %.

### Applications of the maizeSNP3072 array

The maizeSNP3072 array can be used for Chinese maize DNA fingerprinting, germplasm resource evaluation, and molecular marker-assisted breeding. SSRs are currently the primary markers used to identify maize varieties (Wang et al. 2011a). With new developments in molecular technology and increasing identification requirements, SSRs have been found to suffer from various drawbacks such as data sharing and integration problems. These shortcomings can be overcome by using SNP markers. With bead-chip technology, locus throughput ranges from 1 to million SNPs. Because only two alleles exist per SNP, data integration between different laboratories or platforms is easy. The 3072 SNP loci reported in this study were selected on the basis of DNA fingerprinting criteria. The obtained SNP set has high variety-distinguishing efficiency, good reproducibility, and a uniform distribution throughout the genome. The maizeSNP3072 array can be directly applied to maize variety identification, database construction, or genuineness verification using core SNPs selected from the 3072 SNPs. In addition, our assessment of the maizeSNP3072 array demonstrated that the 3072 locus set displayed a superior genotyping performance in inbred lines and, when used in conjunction with a cluster file, could automatically differentiate among three genotypes. The 3072 SNPs showed high levels of polymorphism: 77 % of loci had MAF values >0.30 and 39 % had values >0.40. The maizeSNP3072 array can therefore be used to assess the genetic diversity of maize germplasm resources. Furthermore, the 3072 SNPs can serve as powerful markers for molecular breeding studies, including QTL mapping, background scanning of breeding materials, and homozygosity identification.

## Electronic supplementary material

Supplementary material 1 (PDF 48 kb)

Supplementary material 2 (PDF 357 kb)

Supplementary material 3 (DOCX 13 kb)

Supplementary material 4 (XLSX 14 kb)

Supplementary material 5 (XLSX 12 kb)

Supplementary material 6 (XLSX 403 kb)

Supplementary material 7 (XLSX 1243 kb)

Supplementary material 8 (XLSX 24 kb)
