# Supplementary material for: Development of maizeSNP3072, a high-throughput compatible SNP array, for DNA fingerprinting identification of Chinese maize varieties
Source: Mol Breed. 2015 May 31;35(6):136. doi: 10.1007/s11032-015-0335-0 (PMC4449932; doi:10.1007/s11032-015-0335-0)
Supplement: Supplementary file 1 — Supplementary material 1 (PDF 48 kb) [file 11032_2015_335_MOESM1_ESM.pdf]

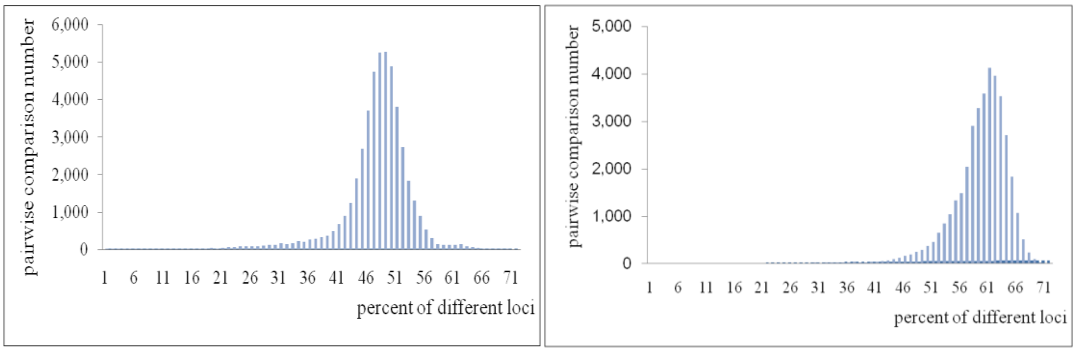

Figure S1 Distribution of different locus percentages obtained by pairwise comparisons of **A** 309 inbreds and **B** 276 hybrids
