# Supplementary figures and images for: Development of maizeSNP3072, a high-throughput compatible SNP array, for DNA fingerprinting identification of Chinese maize varieties
Source: Mol Breed. 2015 May 31;35(6):136. doi: 10.1007/s11032-015-0335-0 (PMC4449932; doi:10.1007/s11032-015-0335-0)

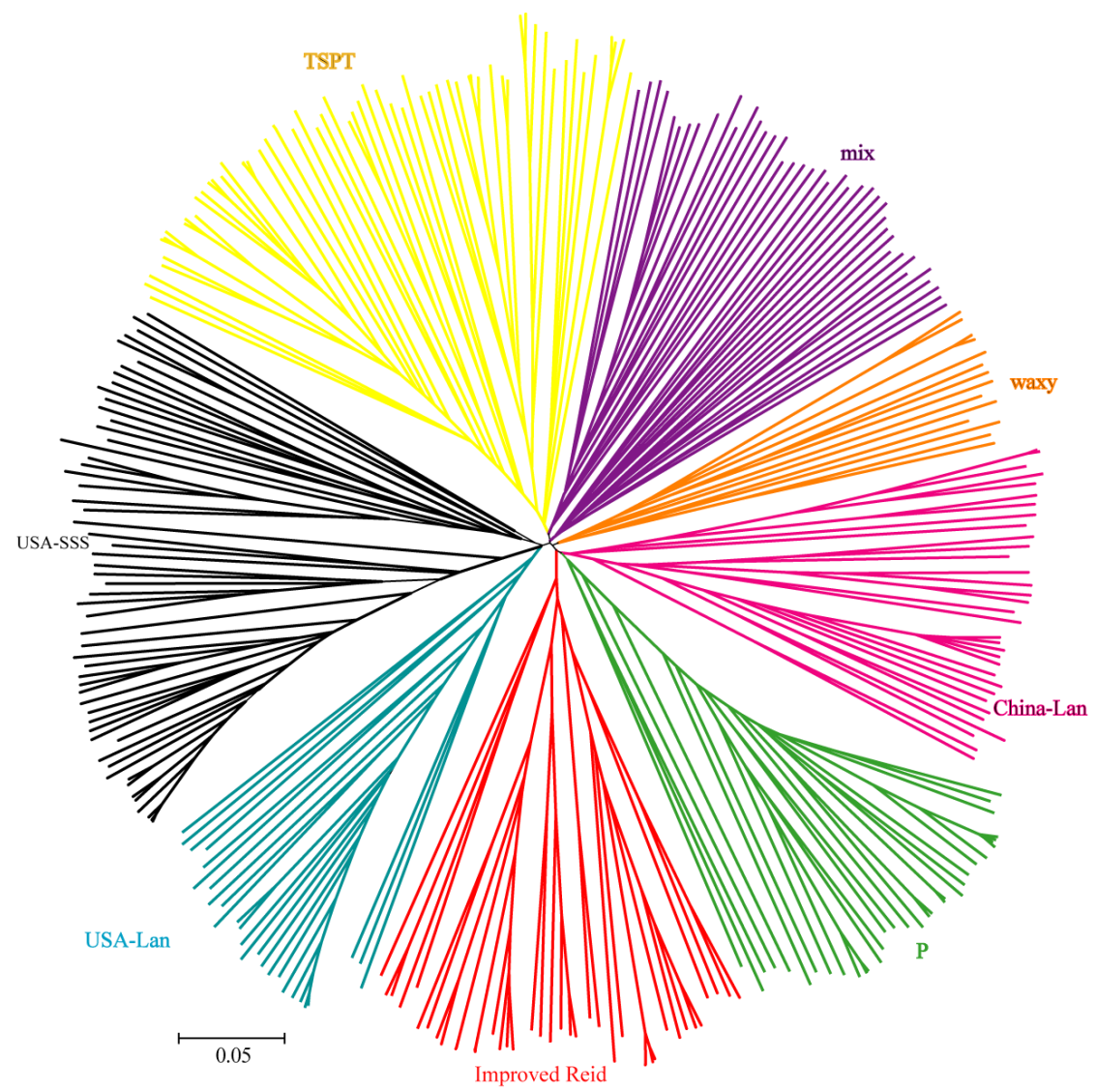

Figure S2 Neighbor-joining (NJ) trees constructed for 309 inbred maize lines based on the 3072 SNPs data

Supplement: Supplementary file 2 — Supplementary material 2 (PDF 357 kb) [file 11032_2015_335_MOESM2_ESM.pdf]
