# Supplementary material for: Development of maizeSNP3072, a high-throughput compatible SNP array, for DNA fingerprinting identification of Chinese maize varieties
Source: Mol Breed. 2015 May 31;35(6):136. doi: 10.1007/s11032-015-0335-0 (PMC4449932; doi:10.1007/s11032-015-0335-0)
Supplement: Supplementary file 3 — Supplementary material 3 (DOCX 13 kb) [file 11032_2015_335_MOESM3_ESM.docx]

**Supplementary materials**

Article title: Development of maizeSNP3072 a high-throughput compatible SNP array for DNA fingerprinting analysis of Chinese maize varieties

Journal name: Molecular Breeding

Author names: Hong-Li Tian* • Feng-Ge Wang* • Jiu-Ran Zhao† • Hong-Mei Yi • Lu Wang • Rui Wang • Yang Yang • Wei Song

H.-L. Tian* • F.-G. Wang* •J.-R. Zhao† • H.-M. Yi • L. Wang • R. Wang • Y. Yang • W. Song

Maize Research Center, Beijing Academy of Agriculture & Forestry Sciences, Beijing Key Laboratory of Maize DNA Fingerprinting and Molecular Breeding, Shuguang Garden Middle Road No. 9, Beijing 100097, China

*Both authors contributed equally to this work.

†Correspondence should be addressed to Jiu-Ran Zhao. E-mail: maizezhao@126.com; Fax: +86-10-51503936; Tel: +86-10-51503936)

**Table S1.** 96 maize samples used for validation of 56,110 single nucleotide polymorphism markers

**Table S2.** Parent/hybrid heritability analysis based on all single nucleotide polymorphisms that were scorable in each hybrid and its two parents

**Table S3.** Detailed information on the 3,072 SNPs reported in this study

**Table S4.** Genotyping data of the 96 maize lines and hybrids used for the selection of SNP loci

**Table S5.** Information on 309 inbred lines used for the validation of 3072 SNP markers

**Figure S1.** Distribution of different locus percentages obtained by pairwise comparisons of **A** 309 inbreds and **B** 276 hybrids

**Figure S2.** Neighbor-joining (NJ) trees constructed for 309 inbred maize lines based on the 3072 SNPs data
